# Global microRNA and protein expression in human term placenta

**DOI:** 10.3389/fmed.2022.952827

**Published:** 2022-10-18

**Authors:** Hanna Östling, Maria Lodefalk, Helena Backman, Robert Kruse

**Affiliations:** ^1^Department of Obstetrics and Gynaecology, Faculty of Medicine and Health, Örebro University, Örebro, Sweden; ^2^Department of Paediatrics, Faculty of Medicine and Health, Örebro University, Örebro, Sweden; ^3^University Health Care Research Center, Faculty of Medicine and Health, Örebro University, Örebro, Sweden; ^4^iRiSC - Inflammatory Response and Infection Susceptibility Centre, Faculty of Medicine and Health, Örebro University, Örebro, Sweden; ^5^Department of Clinical Research Laboratory, Faculty of Medicine and Health, Örebro University, Örebro, Sweden

**Keywords:** fetal growth, inflammatory response, microRNA, placenta, proteomics, RNA-sequencing, term pregnancy

## Abstract

**Introduction:**

Description of the global expression of microRNAs (miRNAs) and proteins in healthy human term placentas may increase our knowledge of molecular biological pathways that are important for normal fetal growth and development in term pregnancy. The aim of this study was to explore the global expression of miRNAs and proteins, and to point out functions of importance in healthy term placentas.

**Materials and methods:**

Placental samples (*n* = 19) were identified in a local biobank. All samples were from uncomplicated term pregnancies with vaginal births and healthy, normal weight newborns. Next-generation sequencing and nano-scale liquid chromatographic tandem mass spectrometry were used to analyse miRNA and protein expression, respectively.

**Results:**

A total of 895 mature miRNAs and 6,523 proteins were detected in the placentas, of which 123 miRNAs and 346 proteins were highly abundant. The miRNAs were in high degree mapped to chromosomes 19, 14, and X. Analysis of the highly abundant miRNAs and proteins showed several significantly predicted functions in common, including immune and inflammatory response, lipid metabolism and development of the nervous system.

**Discussion:**

The predicted function inflammatory response may reflect normal vaginal delivery, while lipid metabolism and neurodevelopment may be important processes for the term fetus. The data presented in this study, with complete miRNA and protein findings, will enhance the knowledge base for future research in the field of placental function and pathology.

## Introduction

The placenta is essential for normal fetal development and growth. It mediates gas exchange and nutritional supply to the fetus as well as removal of waste products. It also produces hormones that regulate maternal physiology in benefit of fetal growth and it acts as an immune barrier (1).

A growing field of interest in placental research concerns the way epigenetic mechanisms impact gene expression in the placenta, thereby affecting placental function. Examining epigenetic alterations in the placenta can provide critical insights into the biology of development and the pathogenesis of diseases (2, 3).

MicroRNAs (miRNAs) are small, non-coding RNA-molecules that act as epigenetic modulators by binding to specific mRNA targets and downregulating gene expression at the posttranscriptional level. Proteins, the final products of gene expression, are structural as well as functional molecules, essential for almost all biological processes in living organisms. Since miRNAs are expressed in all tissues and each miRNA may regulate hundreds of mRNAs, thereby affecting the corresponding protein expression, miRNAs may have an impact on almost all vital organ processes (4, 5).

Previous studies of miRNA or protein expression in the placenta have shown differences in pregnancies complicated by such issues as preeclampsia (6–9), fetal growth restriction (10–12), gestational diabetes (13–15), fetal macrosomia (16), preterm birth (17, 18), and recurrent miscarriages (19, 20). Most previous studies have focused on differences in specific miRNAs, and few have compared differences in global placental miRNA expression (10, 21). While there are several studies of proteomic differences in relations to pregnancy complications, few previous studies have presented the global protein expression in healthy placentas (8, 9, 22). Even less is known about global miRNA expression in healthy placentas, as well as of combined findings on the miRNA expression with an analysis of the protein expression in the same material.

Description of the global expression of miRNAs and proteins in healthy human term placentas can increase the knowledge of molecular biological pathways important for normal fetal growth and development. Defining the normal miRNA signature and its connections to the normal protein expression will also enable the generation of new hypotheses and improve interpretation of future research on placentas from complicated pregnancies.

Therefore, the aim of this study was to explore and describe the global expression of miRNAs and proteins in healthy term placentas, and to describe associated biological functions of possible importance in the placenta.

## Materials and methods

Placental samples were obtained from the biobank at Örebro University Hospital, Örebro, Sweden. The samples used in this study were collected from 2008 to 2012 at the Department of Obstetrics and Gynaecology, Örebro University Hospital, with written informed consent from the women. The Regional Board of Ethics in Uppsala, Sweden (2010/189) approved the study.

The inclusion criteria were healthy women without chronic disease or malabsorption disorder, a normal body mass index (BMI) 18.5–24.9 in early pregnancy, gestational weight gain within the recommended interval of 11.5–16.0 kg (23) and age 18–35 years at the time of delivery. Further, the woman had to give birth vaginally to a healthy, singleton infant with a normal birth weight defined as ±1 SD from the population mean (24) at gestational age 37+0 to 41+6. Maternal exclusion criteria were smoking during pregnancy, any long-term treatment with medicines (excepting folic acid and iron supplements), any pregnancy complications (e.g., gestational hypertension, preeclampsia, gestational diabetes, or erythrocyte immunization, preterm premature rupture of membranes, chorioamnionitis), mechanical induction or induction of labour with prostaglandins, or an active second stage of labour > 60 min. Fetal exclusion criteria were asphyxia (defined as Apgar score < 7 at 5 min), and chromosomal or severe anatomical malformation of the newborn infant.

Nineteen subjects fulfilling all the inclusion criteria and none of the exclusion criteria were identified. These subjects had previously been included as controls in a study focused on miRNA expression in placentas of infants born small for gestational age (10). The characteristics of the nineteen women and infants are presented in Table 1.

**TABLE 1:** Clinical characteristics of the subjects included in the study.

Maternal characteristics (*n* = 19)	
Age (years)	30.0 (23–35)
Parity (0-para)	13 (68.4%)
Early pregnancy weight (kg)	62.7 (55–74)
Height (cm)	167.5 (160–178)
Early pregnancy BMI (kg/m^2^)	22.4 (19.0–24.9)
Gestational weight gain (kg)	14.9 (13–16)

**Newborn infant characteristics (*n* = 19)**	

Gestational age (weeks)	40.1 (37.0–41.7)
Sex (females)	5 (26.3%)
Birth weight (g)	3,702 (3,080–4,355)
Birth length (cm)	51 (48–54)
Birth weight *z*-score	0.03 (–0.87–0.68)
Birth length *z*-score	–0.19 (–1.40–1.37)

Data are presented as medians (min–max) or numbers (%). BMI, body mass index.

Maternal and infant data were retrieved from medical health records. BMI was calculated based on the woman’s measured weight and height in early pregnancy. Gestational weight gain was calculated as the difference between the measured weight of the woman in the delivery ward and the weight in early pregnancy. *Z*-scores for birth weight and birth length of the newborn infants were calculated based on a Swedish reference population, with gestational age and infant sex taken into account (24).

### Sample preparations

The placental samples were collected shortly after delivery. Pieces of approximately 1 cm^3^ were sampled after removal of the decidual layer. Samples were collected from the interior of the placenta, and the marginal portion and the area near cord insertion were avoided as stated before (25–27). The samples were rinsed in 30 mL cold phosphate buffered saline (Gibco, Life Technologies, Stockholm, Sweden) and stored in 3 mL RNAlater (Ambion, Stockholm, Sweden) at –80°C until further processing for RNA and protein extraction.

### RNA isolation

Total RNA was extracted from 10 to 30 mg of each placental sample using the Allprep DNA/RNA/miRNA Universal Kit (28), catalog no. 80224 (Qiagen, Nordic, Sollentuna, Sweden) according to the manufacturer’s instructions. RNA yield and purity were determined with a Nano-Drop ND-1000 Spectrophotometer (Nano-Drop Technology, Wilmington, DE, USA), and RNA quality was evaluated using an Agilent 2100 Bioanalyzer (Agilent Technologies, Palo Alto, CA, USA) according to the manufacturer’s instructions, as previously reported (10). All nineteen samples of RNA were of high quality with an optical density 260/280 ratio of 1.8–2.2 and an RNA integrity number ≥ 8.

### Next-generation sequencing and pre-analytic processing of data

Next-generation sequencing (NGS) was performed at GATC Biotech AG (Konstanz, Germany). Small RNA libraries were generated with Illumina’s Small RNA sample preparation protocol [TruSeq Small RNA Sample Prep Kits (Illumina, San Diego, CA, USA)] according to the manufacturer’s instructions with minor adaptations. The libraries were sequenced using NGS on HiSeq 2500 (Illumina) according to the manufacturer’s protocol, with single read sequencing of > 10 million reads per sample. All pre-analytic processing and analyses of miRNA data were performed using the Strand NGS software suite version 3.3.1.^1^ All reads were subjected to adaptor trimming, followed by trimming of bases with a base quality ≤ 20. Trimmed reads were aligned and mapped to the human reference genome [Genome build: Homo sapiens, HG 19 (build 2009.06.14)] allowing up to one mismatch per uniquely aligned read. DESeq normalization of data was performed prior to statistical and bioinformatic analyses, as previously reported (10).

### Quantitative proteomics analyses using nLC-MS/MS

The placental tissues were prepared and analysed as described by Truvé et al. (29). In brief, tissues were homogenized by bead-beating, and proteins were extracted and digested using trypsin. All samples were labelled using tandem mass tag (TMT). After purification by reverse-phase chromatography using Pierce Peptide Desalting Spin Columns (Thermo Fisher Scientific, Waltham, MA, USA), peptides were fractionated by basic reversed-phase liquid chromatography (bRP-LC) and subsequently analysed by nano liquid chromatography mass spectrometry (nLC-MS/MS) on an Orbitrap Fusion™ Tribrid™ mass spectrometer (Thermo Fisher Scientific) at the Proteomics Core Facility, Gothenburg University, Sweden. Identification and relative quantification were performed using Proteome Discoverer version 2.3 (Thermo Fisher Scientific). The database search was performed using the Mascot search engine v. 2.5.1 (Matrix Science, London, UK) matching with the SwissProt *H. sapiens* database (September 2019). For quantification, TMT reporter ions were identified in the MS3 higher-energy collision dissociation (HCD) spectra, and the TMT reporter intensity values for each sample were normalized within Proteome Discoverer on the total peptide amount. Only the unique peptides were taken into account for the relative quantification. Data of proteins passing a protein false-discovery rate of 5% were exported for further processing in Microsoft Excel.

### Statistical and bioinformatic analyses

Maternal and infant characteristics are presented as medians (min–max) or numbers (%). Explorative data and statistical analysis of miRNA and protein expression were performed in Strand NGS and GeneSpring GX version 11.5 (Agilent Technologies, Palo Alto, CA, USA), respectively. Comparisons between sexes were performed with Welch’s *t*-test followed by Benjamini-Hochberg’s method for multiple testing correction. Significance for gene ontology (GO) term enrichment was analysed in Strand NGS with GO accessions of potential mRNA targets for miRNAs, and in GeneSpring GX for proteins. Ingenuity Pathway Analysis (IPA, QIAGEN Inc.) core analyses were used for functional bioinformatics analyses of miRNAs and proteins with respect to upstream regulators, downstream biological functions and interaction networks. The individual cut-offs for abundancy of miRNAs and of proteins for downstream analyses were set differently. For miRNAs the cut-off was in general set to a more stringent level in order to focus on highly expressed miRNAs and their impact on placenta, while a lower cut-off was set for proteins in order to gain focus on entities beyond highly expressed structural proteins.

### Linking of microRNAs to proteins

Cytoscape (version 3.9.1) and the CyTargetLinker plug-in (version 4.1.0) were used to link miRNAs to proteins (target gene products) with miRTarBase (release 8.0) entries. Expression levels of miRNA and proteins were for visualisation in Cytoscape z-score normalised separately within each dataset such that the mean of all of the values within each dataset was set to *z*-score 0. miRNAs with a normalised value below zero were removed in the Cytoscape visualisations in order to focus on moderately to highly expressed miRNAs in placenta. In addition, miRNAs and proteins with only one or no edge between a miRNA and a protein were removed in the visualisations. The stringApp (version 1.7) plug-in in Cytoscape was used to retrieve and filter for GO and reactome (HSA) pathway annotations for the targeted gene products (proteins).

## Results

In order to describe the global miRNA and protein expression in human term healthy placenta, we both show the 20 most highly expressed individual entities for miRNAs and proteins respectively, as well as predicted regulators and biological functions of a larger number of highly expressed entities. Finally, we present interactions between expressed miRNAs and proteins in a network analysis.

### MicroRNA expression

Aligned reads from the placental samples were annotated to 895 unique miRNA sequences (miRBase accessions). Of these, 270 miRNA sequences were highly abundant (defined as being present in ≥ 90% of the samples at an expression level of ≥ 90th percentile of total reads in each sample). In IPA, these 270 miRNA sequences functionally mapped to 123 unique miRNAs (disregarding loci and stem loop origin) (Supplementary Table 1).

**FIGURE 1:**
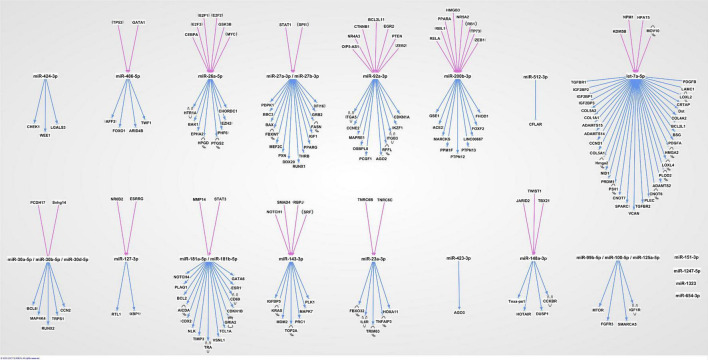
The 20 most highly expressed miRNAs in human term healthy placentas and their predicted enrichment of associated upstream regulators and downstream molecules in IPA knowledge base.

**FIGURE 2:**
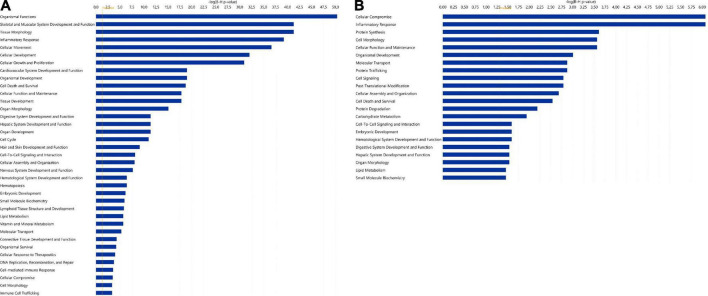
Significantly enriched functions for **(A)** the 123 highly expressed miRNAs and **(B)** the 346 highly expressed proteins in human term healthy placentas. Data presented as –log (*p*-value), *p* = 0.05. For both analyses, diseases and disorders have been excluded. In panel **A**, the function “gene silencing/gene expression” is not shown but, as expected, it was the most prominent function.

The 20 miRNAs with the highest expression in all samples and their known predicted upstream regulators and downstream effector molecules, are presented in Figure 1. More than half of the most predicted upstream regulators of all the highly abundant 123 miRNAs were connected to transcriptional processes (Table 2). The downstream function analysis showed 37 significantly predicted functions, including inflammatory response, basic cell biology and development and function of different organ systems (Figure 2A).

**TABLE 2:** Top 20 upstream regulators of the 123 highly expressed miRNAs in human term healthy placentas.

Gene symbol	Gene name/Encoded protein	Function	*P*-value
*SSB*	Small RNA Binding Exonuclease Protection Factor La	Enzyme	8.49E-78
*EPHB6*	Ephrin Type-B Receptor 6	Kinase	6.32E-40
*GNAS-AS1*	GNAS Antisense RNA 1	Non-protein coding	1.70E-35
*TP53*	Tumor Protein P53	Transcription regulator	6.32E-25
*TNFRSF1B*	Tumor Necrosis Factor Receptor Superfamily Member 1B	Transmembrane receptor	5.77E-22
*Smad2/3*	SMAD Family Member 2 and 3	Signal transducers and transcriptional modulators	2.21E-20
*BRAF*	B-Raf Proto-Oncogene, Serine/Threonine Kinase	Kinase	1.80E-18
*TMEM8B*	Transmembrane Protein 8B	Probable tumor suppressor	8.08E-18
*NF2*	Neurofibromin 2	Interact with cell-surface proteins, proteins involved in cytoskeletal dynamics and proteins involved in regulating ion transport.	1.83E-16
*IGF1R*	Insulin Like Growth Factor 1 Receptor	Transmembrane receptor	2.98E-14
*E2F3*	E2F Transcription Factor 3	Transcription regulator	1.07E-13
*PPARA*	Peroxisome Proliferator Activated Receptor Alpha	Ligand-dependent nuclear receptor	4.68E-12
*ERBB2/HER2*	Erb-B2 Receptor Tyrosine Kinase 2	Kinase	7.57E-12
*APC*	APC Regulator of WNT Signaling Pathway	Enzyme	3.62E-11
*MAP2K1/2*	Mitogen-Activated Protein Kinase Kinase 1 and 2	Essential components of the MAPK/ERK signaling pathway	1.14E-10
*Insulin*	Insulin	Peptide hormone	1.43E-10
*c-Src*	SRC Proto-Oncogene, non-Receptor Tyrosine Kinase	Proto-oncogene	1.57E-10
*INSR*	Insulin Receptor	Kinase	1.70E-10
*TGFB1*	Transforming Growth Factor Beta 1	Growth factor	1.89E-10
*MTDH*	Metadherin	Transcription regulator	3.56E-10

The loci for the highly abundant miRNAs were mapped to all human chromosomes except chromosomes 18 and Y. The most prominent mapping was to chromosome 19 (24%), followed by chromosome 14 (10%), chromosome X (9%), chromosome 17 (7%), and chromosome 1 (7%).

### Protein expression

A total of 6,523 proteins were detected, of which 3,773 were expressed in ≥ 90% of the samples. Of these, 346 proteins had an expression level of ≥ 50th percentile of total peptide amount in each sample (Supplementary Table 2).

Among the 20 proteins with the highest expression in ≥ 90% of the samples, were proteins involved in lipid metabolism (DBI, PEX19, LRPAP1, CD36), immune response (RNASET2, GGH, CD209, CTSC), and neuronal growth and development (GAP43, LZTFL1, ANP32E), (Table 3). As for miRNAs, several of the most significantly predicted upstream regulators of the highly abundant proteins were found to be known transcriptional regulators (Table 4). In the downstream biological function analysis 16 out of 22 predicted functions for proteins were also predicted in the miRNA function analysis; cellular compromise, inflammatory response, cell morphology, cellular function and maintenance, organismal development, molecular transport, cellular assembly and organization, cell death and survival, cell-to-cell signalling an interaction, embryonic development, haematological system development and function, digestive system development and function, hepatic system development and function, organ morphology, lipid metabolism, small molecule biochemistry (Figure 2B).

**TABLE 3:** The 20 most highly expressed proteins in human term healthy placentas.

Protein symbol	Gene name/Encoded protein	Function	Expression intensity
SMAGP	Small cell adhesion glycoprotein	May play a role in epithelial cell-cell contacts.	2.17
ARSA	Arylsulfatase A	Enzyme	2.17
COX17	Cytochrome C oxidase copper chaperone COX17	Enzyme. The terminal component of the mitochondrial respiratory chain.	1.98
DBI	Diazepam binding inhibitor, Acyl-CoA binding protein	Lipid metabolism	1.96
PEX19	Peroxisomal biogenesis factor 19	Peroxisomal biogenesis	1.95
GAP43	Growth associated protein 43	Considered a component of regenerative response in the nervous system.	1.91
RNASET2	Ribonuclease T2	Enzyme. Plays an essential role in innate immune response.	1.85
GGH	Gamma-glutamyl hydrolase	Peptidase. Among related pathways are glutathione metabolism and innate immune system.	1.80
LRPAP1	LDL receptor related protein associated protein 1	Interacts with the low density lipoprotein (LDL) receptor-related protein.	1.77
LZTFL1	Leucine zipper transcription factor like 1	Regulates protein trafficking to the ciliary membrane. May play a role in neurite outgrowth.	1.76
ANP32E	Acidic nuclear phosphoprotein 32 family member E	Histone chaperone. May be of importance in cerebellar development and synaptogenesis.	1.76
CD36	CD36 molecule	Transmembrane receptor	1.74
MRRF	Mitochondrial ribosome recycling factor	Component of mitochondrial translation.	1.75
ANP32A	Acidic nuclear phosphoprotein 32 family member A	Involved in several cellular processes, including proliferation, differentiation, apoptosis, and tumor suppression.	1.732
CD209	CD209 molecule	Pathogen-recognition receptor, involved in initiation of primary immune response.	1.73
CCDC58	Coiled-coil domain containing 58	Other	1.72
TXNDC9	Thioredoxin domain containing 9	Associated with cell differentiation.	1.72
CTSC	Cathepsin C	Peptidase	1.71
CKAP4	Cytoskeleton associated protein 4	Mediates the anchoring of the endoplasmic reticulum to microtubules.	1.70
THOC7	THO complex 7	Among related pathways are cleavage of growing transcript and transport of mature transcript to cytoplasm.	1.70

**TABLE 4:** Top 20 upstream regulators of the 346 highly expressed proteins in human term healthy placentas.

Gene symbol	Gene name/Encoded protein	Function	*P*-value
*HNF4A*	Hepatocyte Nuclear Factor 4 Alpha	Transcription regulator	1.27E-14
*RICTOR*	RPTOR Independent Companion Of MTOR Complex 2	Component of a protein complex that integrates nutrient- and growth factor-derived signals to regulate cell growth.	2.98E-09
*miR-155-5p*	microRNA 155-5p (miRNAs w/seed UAAUGCU)	Mature microRNA	1.70E-35
*CLN3*	Ceroid-Lipofuscinosis, Neuronal 3	Mediates microtubule-dependent transport.	7.59E-07
*CAB39L*	Calcium Binding Protein 39 Like	Kinase	1.69E-06
*KDM5A*	Lysine Demethylase 5A	Transcription regulator	6.41E-06
*PDGF (family)*	Protein family comprised of both platelet-derived growth factors (PDGF) and vascular endothelial growth factors (VEGF).	Essential for regulation of embryonic development, cell proliferation, cell migration, cell survival and chemotaxis.	6.72E-06
*MAPT*	Microtubule Associated Protein Tau	Promotes microtubule assembly and stability.	1.07E-05
*NRF1*	Nuclear Respiratory Factor 1	Transcription regulator	1.13E-05
*LONP1*	Lon Peptidase 1, Mitochondrial	Peptidase	2.55E-05
*TLE3*	Transducin-Like Enhancer Protein 3	Transcriptional co-repressor	5.70E-05
*QKI*	Quaking Homolog, KH Domain RNA Binding	RNA-binding protein that regulates pre-mRNA splicing, export of mRNAs from the nucleus, protein translation, and mRNA stability.	3.00E-04
*MYC*	MYC Proto-Oncogene, BHLH Transcription Factor	Transcription regulator	3.79E-04
*H1-6*	H1 Histone Family, Member T	Histone	3.81E-04
*H1f1*	H1 Histone Family, Member 1	Histone	3.81E-04
*DDX5*	DEAD-Box Helicase 5	Enzyme	4.67E-04
*ONECUT1/HNF6*	One Cut Homeobox 1/Hepatocyte Nuclear Factor 6	Transcription regulator	4.93E-04
*HBA1/HBA2*	Hemoglobin Subunit Alpha 1/2	Transporter	5.75E-04
*RB1*	RB Transcriptional Corepressor 1	Transcription regulator	5.90E-04
*HNRNPH1*	Heterogeneous Nuclear Ribonucleoprotein H1	Appear to influence pre-mRNA processing and other aspects of mRNA metabolism and transport.	6.38E-04

The highly expressed proteins were further categorized according to biological function and cellular localization. The most prominent biological functions were enzymes (23%), transporters (10%), and transcription regulators (8%). The proteins were located to cytoplasm (61%), nucleus (18%), plasma membrane (12%), and extracellular space (3%).

### Network of microRNAs and proteins expressed in placenta

**FIGURE 3:**
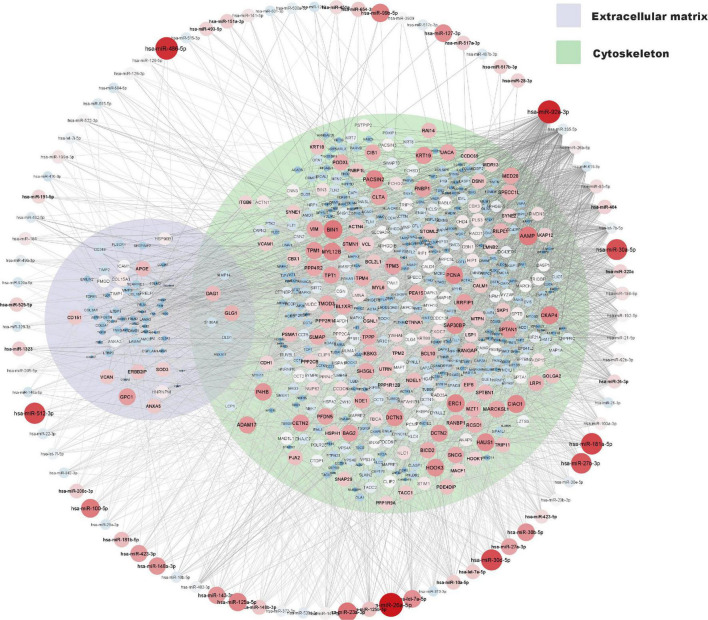
miRNA and protein expression levels annotated to structural integrity in placenta. Normalised expression levels of miRNAs with a *z*-score above zero and proteins are illustrated on a continuous scale from lowest expression (bluer smaller nodes) to highest expression (redder larger nodes) and the miRNA target links (edges) to gene products sorted with respect to their degree of edges. Proteins annotated to cytoskeleton (GO: 0005856) and or extracellular matrix (GO: 0031012) are shown within their respective Venn-circular areas.

**FIGURE 4:**
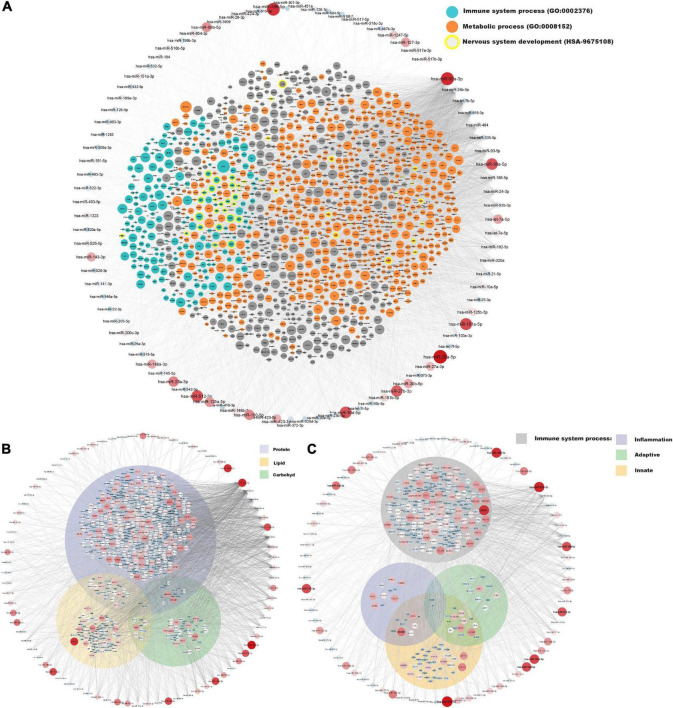
miRNA and protein expressions annotated to immune system and metabolic processes in placenta. Normalised expressions of miRNAs with a *z*-score above zero and proteins are illustrated on a continuous scale from lowest expression (bluer smaller nodes) to highest expression (redder larger nodes) and the miRNA target links (edges) to gene products sorted with respect to their degree of edges. **(A)** All proteins expressed in at least 90% of placenta samples (structural proteins in Figure 3 have been removed) are shown with focused annotations to immune system process (turquoise) and metabolic process (orange) gene ontologies and nervous system development (yellow border) reactome pathway, denoted by node colour. Majority of turquoise nodes are also annotated to metabolic process (not shown). **(B)** Proteins annotated to metabolic process further annotated to specific metabolic processes [Protein metabolic process (GO: 0019538), Lipid metabolic process (GO: 0006629), and Carbohydrate metabolic process (GO: 0005975)] shown within their respective Venn-circular areas. **(C)** Proteins annotated to immune system process further annotated to sub-immune processes [Inflammatory response (GO: 0006954), Innate immune response (GO: 0045087), and Adaptive immune response (GO: 0002250)] shown within their respective Venn-circular areas.

A network of moderately to highly expressed miRNAs and their linked proteins expressed in our samples and annotated to structural integrity in placenta is shown in Figure 3. After exclusion of these structural proteins, miRNAs linked to all remaining proteins (expressed in ≥ 90% of placental samples) are shown in Figure 4A, highlighting annotations to the immune system, metabolic processes and nervous system development. Proteins annotated to metabolic processes were further divided into protein, lipid and carbohydrate metabolism (Figure 4B). Proteins annotated to the immune system were divided into inflammatory response, innate immune response and adaptive immune response (Figure 4C).

### Immune and inflammatory annotations

As we found that the inflammatory response was an important predicted function for both miRNAs and proteins expressed in our samples, we investigated connections to the immune system in more detail. Of the 3,773 proteins expressed in ≥ 90% of the samples and the 123 highly abundant miRNAs, 130 proteins and 14 miRNAs were annotated to the biological function “immune response of cells.” Of them, 32 proteins and seven miRNAs were annotated to decreased function, and 82 proteins and four miRNAs were annotated to increased function, according to publications supporting the gene-to-function relationship in IPA. Further, 249 proteins and 50 miRNAs were annotated to “inflammation of organ.” Of them, 77 proteins and three miRNAs were annotated to decreased function, and 45 proteins and four miRNAs were annotated to increased function, according to publications. The complete list for the miRNAs and proteins annotated to immune and inflammatory response is shown in Supplementary Table 3.

### Fetal sex

As fetal sex may impact gene expression in placental tissue, we investigated whether there were any sex differences in our material. Two miRNAs, miR-4661-5p, and miR-371a-3p, were significantly upregulated [fold change (FC) = 3.2; *p* = 0.019 and FC = 3.0; *p* = 0.01, respectively] and one protein, DDX3Y, was significantly downregulated (FC = –11.4; *p* = 0.008) in placentas originating from females (*n* = 5) compared to males (*n* = 14). No other significant differences were seen with regard to fetal sex.

## Discussion

In this study, we described the global expression of miRNAs and proteins in human term placentas of uncomplicated pregnancies. In total 895 mature miRNAs and 6,523 proteins were detected in the placentas using NGS and nLC-MS/MS. Previous omic-studies of placental miRNA and protein expression have shown a large variation in numbers of detected entities (8, 9, 21, 22, 30). These differences may be due to several factors, including differences in the study population, methods of laboratory analysis and access to more complete miRNA and protein library databases over time.

The miRNAs in the present study were in high degree mapped to gene loci on chromosomes 19, 14, and X. This is consistent with the findings of Carreras-Badosa et al. (21). It is also consistent with previous knowledge of three miRNA clusters on chromosomes 19 and 14 with placenta-specific expression; the chromosome 19 miRNA cluster (C19MC), the chromosome 14 miRNA cluster (C14MC) and the miR-371-3 cluster, also situated on chromosome 19 (31). There is evidence that the expression patterns of these miRNA clusters may vary throughout pregnancy, with decreasing expression of the C14MC from the first to the third trimester, though data for the C19MC and the miR-371-3 cluster have shown conflicting results (32, 33). The C19MC miRNAs have been shown to be the predominant miRNA species in term human trophoblasts (34), consistent with our findings. In the present study, other miRNA clusters on chromosome 19 and 14 were also represented in the material. These clusters are less well described in pregnancy and placentas. One of these clusters that includes miR-23a, miR-24, and miR-27a, which were all highly expressed in our samples, is mapped to chromosome 19 and connected to the biological function inflammatory response. Previous studies of these three miRNAs in placentas or pregnancy have shown that miR-23a and miR-27a may impair trophoblast migration and invasiveness (35, 36). Upregulation of miR-24 has been associated with preeclampsia (37). Even though this miRNA cluster is not known to be placenta-specific, it could still be of interest for a deeper understanding of normal mechanisms in term pregnancy and parturition. The most abundant miRNAs in the placental samples of the present study were in high degree mapped to chromosome X. This may be a result of the normal genomic distribution where the human X chromosome has a higher density of miRNA genes compared to the average autosomes (38). To date most of the X-linked miRNAs have no described function. However, previous studies have shown that the chromosome X in mammalians is of importance in immune functions and autoimmunity and may contribute to sex differences seen in cancer and inflammatory diseases (39, 40). It is not well documented whether the high amount of miRNAs on chromosome X also reflects these immunological functions, though there is emerging evidence of that (41). Since we found immune response and inflammation to be important predicted functions in the placentas of our study, the high proportion of miRNAs linked to chromosome X may reflect these biological functions and not only be explained by the high density of miRNA genes on the chromosome X *per se*.

In the present study, enzymes were the most abundant protein category in the material, and the largest proportion of detected proteins were predicted to the cytoplasmic compartment of cells, consistent with previous findings by Mine et al. (8). Among the 20 proteins with the highest expression were proteins involved in lipid metabolism, immune response and neuronal growth and development (see Table 3). Nervous system development and function was also found to be a predicted function in the downstream analysis of the 123 miRNAs, and inflammatory response and lipid metabolism were seen to be predicted downstream functions for both the highly expressed proteins and miRNAs (see Figures 2A,B). Further, when investigating the miRNAs connections to all proteins expressed in ≥ 90% of placental samples (structural proteins excluded), annotations to metabolic processes, immune response processes and nervous system development were highly represented (Figure 4A).

Our results are partially supported by previous protein studies, where metabolic pathways and inflammatory processes have been demonstrated as important functions in placenta. Mushahary et al. showed that cell stress, metabolism and cytoskeleton were among the most common functions for the proteins in their placental samples (22). Since we chose to exclude structural proteins from the functional analysis, we cannot expect to find cytoskeleton as a predicted function, but it is plausible that there is an overlap in the functions cell stress and inflammatory response. Wang et al. in a comparative study of placentas of normal versus pre-eclamptic pregnancies, showed that metabolism was the most important function for all proteins in their material, and together with immune system processes and cell differentiation among the top three networks involved in the differentially expressed proteins (9). This result is consistent with our findings, even though Wang et al. investigated placentas after caesarean section, while the placentas in our study were collected after vaginal delivery. For miRNA, the information about global expression patterns and predicted pathways in human placenta is sparse. Gu et al. comparing miRNA expression in first and third trimester placentas, showed that miRNAs involved in innate and adaptive immune responses were highly expressed in both trimesters, whereas miRNAs involved in cell differentiation and tumour suppression were predominantly expressed in late pregnancy (33). Guo et al. investigating placental miRNA expression variance with focus on demographic factors in a normal pregnant population, concluded that population affiliation and fetal sex may influence miRNA expression, but did not specify any commonly predicted functions in their material (30), nor did Carreras-Badosa et al. in their comparative study of global miRNA expression in correlation to maternal weight during pregnancy (21). In a transcriptomic study by Majewska et al. transcriptional processes and innate immune response were among the most predicted biological functions in placentas of healthy women, following elective caesarean section (42). Winn et al. showed that transcription, lipid metabolism and immune response were among the most significantly annotated functions for the differently expressed genes in term placentas compared to midgestation placentas (43). Further, Saben et al. showed that placenta-enriched genes were connected to functional processes involving adaptive immunity and immune response, energy metabolism and insulin signalling (44). While miRNA expression may be included in transcriptomic studies, the correlation between mRNA and protein expression data is moderate, and so comparison between transcriptomic and proteomic studies should be done with care. However, we think that our study, including both miRNA and protein expression data for analysis of predicted biological functions, adds complimentary information to these previous transcriptional studies.

Inflammation is a coordinated response of the immune system that can be activated by a variety of factors, including cell damage and pathogens. The miRNAs and proteins annotated to the biological functions “immune response of cells” and “inflammation of organ” in the present study were in equal proportions annotated to either increased or decreased functions. These annotations suggest that immune response and inflammation is normal physiological processes in healthy placentas, characterized by a balance between pro-inflammatory and anti-inflammatory signals. This interpretation is supported by previous knowledge of inflammatory response being vital for normal placental development and function, from implantation to delivery (45). The placentas in the present study were all collected after vaginal labour, which may have affected the result. Term parturition is characterized by sterile inflammation with an increase in inflammatory markers seen in various feto-maternal tissues (46–48). However, transcriptomic studies of inflammatory response in laboured versus non-laboured placentas have shown conflicting results (28, 49–51). Further, a prolonged pushing phase is a well-known risk factor for adverse maternal and fetal outcomes such as fetal hypoxia. A recent study from our group found an inverse association between duration of the pushing phase and placental gene expression (52). These findings indicate that duration of pushing should be taken into account in studies of laboured placentas. Thus, to improve the reliability of our results in the present study, we excluded women with a prolonged pushing phase (> 60 min).

The predicted function lipid metabolism in term placentas is also of interest as human infants are born with a high proportion of body fat, an energy deposit that is established during the last trimester of pregnancy when fetal weight gain is maximal. Fatty acids are derived from maternal deposits and maternal diet, and are transferred across the placenta (53). In normal third trimester pregnancy, physiological insulin resistance in the pregnant woman promotes maternal lipolysis and increased levels of triglycerides and free fatty acids in maternal blood (54). Fatty acids taken up by the placenta are used in both the placenta’s own metabolism and for further transfer to the fetus. Placental transfer of fatty acids is a complex process that involves several carriers and lipases, although the exact mechanisms are still not fully known (54) and further research is needed in this field. As key nutrients for the fetus, fatty acids are used as a source of energy, as structural components in cell membranes and as precursors for, for example, eicosanoids and peroxisome proliferator activated receptor (PPAR) ligands (55). Interestingly, *PPAR*α was among the most predicted up stream regulators for the highly expressed miRNAs in the present study, which further emphasizes the connection to lipid metabolism.

Essential fatty acids are also of profound importance for normal fetal neuronal growth and development (56). While most fetal organ systems are formed in the first trimester, the neuronal system continues to develop throughout pregnancy and afterward. In the third trimester of pregnancy, there is accelerated synapse formation, increased neuronal differentiation, and an expansion and structural formation of the cerebral cortex (57). The human brain consists of almost 60% fat, and the rapid growth and development of the fetal brain in late pregnancy is dependent on sufficient supply of substrates. Impaired placental transfer of essential fatty acids is associated with impaired fetal neural development (55). Children born preterm, and therefore lacking the normal fat accretion from late pregnancy, have an increased risk of neurodevelopmental delay (58). Further, there is evidence indicating that longer gestation is beneficial to brain development and cognitive functions even among term-born children (59). Thus, the predicted function lipid metabolism found in the present study may not only be connected to increased weight gain of the fetus in general, but may also, together with the predicted function of nervous system development, mirror the increased demand from the expanding fetal nervous system. However, mechanisms involved in placental lipid metabolism and fetal neuronal development need further investigations.

We found differential expression between sexes for two miRNAs, miR-371a-3p, and miR-4661, and for one protein, the sex-specific protein DDX3Y. miR-371a-5p has been shown previously to be upregulated in male placentas (30, 60), whereas miR-371a-3p has been found to be upregulated in female placentas (60), which is in accordance with our finding. miR-371 belongs to the miR-371-3 cluster on chromosome 19, as previously mentioned, which is predominantly expressed in the placenta. miR-371-members are thought to be important for placental development, but have also shown to be differentially expressed in several cancers (31). For miR-4661, there are only few previous publications. It may be involved in ovarian cancer (61), but there are no previous reports of sex-associated expression pattern and its role in placental development is not yet known. However, due to the small sample size in the present study our results should be interpreted with caution.

When defining normality in pregnancy, it is important to consider variables such as obesity (21), smoking (62), gestational age (43), and mode of delivery (46), which all may influence the placental expression and therefore should be reported in studies of gene or protein expression (63). One strength of the present study was the possibility of including strictly selected samples and excluding some potential confounding factors. Despite this, there may be risk factors or confounders that we are not aware of, but we still believe that the result of our study is reasonably representative for healthy term placentas. Further strengths of this study were the use of nLC-MS/MS for large-scale quantitative mapping of proteins and the decision to analyse the 50th percentile of all proteins expressed in ≥ 90% of the placentas. This approach increased the proportion of low abundance proteins included in the bioinformatics, which is preferable since proteins with low abundance can still exert important biological functions. Further, in the biological function analysis of this study, a majority of predicted functions for proteins were also predicted in the miRNA function analysis, including inflammatory response, development of organ systems and metabolism, a concordance between miRNAs and proteins that strengthens the findings.

One limitation of the present study was the small sample size, which was mostly due to the strict selection of subjects in order to ensure normality as far as possible. However, most previous studies of global miRNA or protein expression in the placenta have had similar sample sizes (8, 9, 21, 22), and a recent review of placental transcriptomic studies by Yong et al. showed that the median number of placentas profiled per study was 13 (64). In addition, the placentas in this study were collected after vaginal labour, and so the miRNA and protein expression may have been influenced by the parturition process, as previously discussed (28, 49–51). This must be taken into account when comparing the results of this study with other placental studies where placentas after elective caesarean section were used. Further, as in almost all previous studies of human placentas, the tissues used in this study were collected *ex vivo*. Even if the sampling was performed shortly after delivery, biological processes may have been affected in varying degrees by the hypoxic *ex vivo* milieu. Another limitation, due to the use of whole-tissue, is that it is not possible to specify which cell types that have contributed to the main results, nor to exclude the possible impact of maternal blood contamination. Future *in vitro* omics studies of normal term trophoblasts for confirmative functional pathway analysis are therefore of interest. Finally, software algorithms are limited in their possibility to predict targets with full reliability, and biological validation of critical targets would have been preferred. However, as we only aimed to describe the overall pattern of global miRNA and protein expression, we chose not to validate specific potential targets. In a previous study from our group of miRNA expression in placenta, using the same miRNA analysis platform, technical validation confirmed the results (10).

In conclusion, in this study we present global miRNA and protein expression in a healthy human term placenta cohort. Our findings of highly abundant miRNAs and proteins connected to immune and inflammatory response, lipid metabolism and nervous system development and function are consistent with previous knowledge of fetal growth and development and placental function in late pregnancy. The data presented in this study, including Supplementary material with complete miRNA and protein findings, will contribute to the biomolecular reference base for future studies in the field of placental origin of health and disease.

## Data availability statement

The datasets presented in this study can be found in online repositories. The names of the repository/repositories and accession number(s) can be found below: https://www.ncbi.nlm.nih.gov/geo/, GSE211791, http://www.proteomexchange.org/, project name: Global microRNA and protein expression in human term placenta; accession number: PXD036306.

## Ethics statement

The studies involving human participants were reviewed and approved by the Regional Board of Ethics in Uppsala, Sweden. The patients/participants provided their written informed consent to participate in this study.

## Author contributions

HÖ, RK, and ML designed the work. HÖ selected the subjects, designed the tables, and drafted and wrote the manuscript. RK performed the bioinformatic analyses and designed the figures. HÖ, RK, ML, and HB analyzed the RNA sequence and proteomic data. All authors read and critically revised the manuscript as well as approved the final version of the manuscript.

## Footnotes

1
www.strand-ngs.com

## Supplementary material

Supplementary Table 1 Highly expressed 123 miRNAs in human term healthy placentas.

Supplementary Table 2 Highly expressed 346 proteins in human term healthy placentas.

Supplementary Table 3 miRNAs and proteins annotated to immune and inflammatory response.
